# The empirical codon mutation matrix as a communication channel

**DOI:** 10.1186/1471-2105-15-80

**Published:** 2014-03-22

**Authors:** Dawit Nigatu, Attiya Mahmood, Werner Henkel

**Affiliations:** 1 School of Engineering and Science, Jacobs University Bremen, Campus Ring 1, D-28759 Bremen, Germany

## Abstract

**Background:**

A number of evolutionary models have been widely used for sequence alignment, phylogenetic tree reconstruction, and database searches. These models focus on how sets of independent substitutions between amino acids or codons derive one protein sequence from its ancestral sequence during evolution. In this paper, we regard the Empirical Codon Mutation (ECM) Matrix as a communication channel and compute the corresponding channel capacity.

**Results:**

The channel capacity of 4.1875 bit, which is needed to preserve the information determined by the amino acid distribution, is obtained with an exponential factor of 0.26 applied to the ECM matrix. Additionally, we have obtained the optimum capacity achieving codon distribution. Compared to the biological distribution, there is an obvious difference, however, the distribution among synonymous codons is preserved. More importantly, the results show that the biological codon distribution allows for a “transmission” at a rate very close to the capacity.

**Conclusion:**

We computed an exponential factor for the ECM matrix that would still allow for preserving the genetic information given the redundancy that is present in the codon-to-amino acid mapping. This gives an insight how such a mutation matrix relates to the preservation of a species in an information-theoretic sense.

## Background

Markov models for the protein sequence evolution have been widely used for the past 40 years. These evolutionary matrices highlight the most common mutational changes between amino acids and codons. Protein sequence evolution has been investigated at both amino acid and codon levels. The evolutionary matrices on the basis of amino acids are widely used for sequence alignments and phylogenetic tree construction. As more than one codon encode to the same amino acid, it is easy to estimate alignments in amino acids as compared to codons.

Codon-level models demonstrate the mutational changes among the codons. This gives us more information by highlighting the tendency of mutations between codons encoding the same amino acid (synonymous changes) as well as the mutational effects between codons that code for different amino acids (non-synonymous changes). As codons are the smallest genetic information unit in protein-encoding regions, it is obvious to model mutations by a codon-based communications channel model highlighting all codon-to-codon changes in nature.

Substitution matrices define the rate at which one amino acid in the protein sequence is changed into another amino acid. Dayhoff et al. [1] estimated the first such model in 1972, resulting in the widely used point accepted mutations (PAM) matrix. It is computed by counting the mutations in the closely related proteins. Henikoff and Henikoff proposed the block substitution matrix (BLOSUM) for divergent protein sequences, which uses log-likelihood ratios to construct scoring matrices from the transition matrices between amino acids [2]. Later on, Whelan and Goldman (WAG) proposed a novel approach to estimate amino acid replacement matrices from a large database of aligned protein sequences in 2001 [3]. It combines the estimation of transition and scoring matrices by a maximum-likelihood approach that accounts for the phylogenies of sequences within each training alignment.

As the codon (a tri-nucleotide) is the basic genetic information that directly encodes the amino acid as the building block of proteins, we have used the first empirical codon substitution matrix (ECM) in our analysis. This was proposed by Schneider et al., where sequences of five vertebrates were aligned and the number of codon substitutions were counted among them [4]. According to conversations with the authors, it is estimated that these mutations on average happened in roughly 300 Million years.

Yockey was one of the first to model and describe a central dogma using information theoretic tools [5]. He viewed the flow of information from DNA or RNA to proteins as a communication system and employed entropy, rate, and capacity calculations with a transition matrix he developed by considering base changes of equal probability. A detailed analysis of the application of information theory to molecular biology can be found in his book [6]. Relatively recently, L. Gong, N. Bouaynaya, and D. Schonfeld have proposed a communication model for protein evolution [7]. They used the amino acid based PAM matrix and a matrix they produced, similar to Yockey’s, as a communication channel and performed capacity calculations over it.

We computed an exponential factor for the ECM matrix that would still allow for preserving the genetic information given the redundancy that is present in the codon-to-amino acid mapping. This gives an insight on how such a mutation matrix relates to the preservation of a species in an information-theoretic sense.

For the underlying capacity computation, we used the Arimoto-Blahut algorithm [8,9] to determine the input distribution that maximizes the mutual information.

## Methods

In order to compute the mutation probability in the ECM matrix, 17502 alignments of sequences from five vertebrate genomes yielded 8.3 million aligned codons from which the number of substitutions between codons were counted. This matrix has 64×64 entries stating the mutation probability of each codon to every other codon. Basically, the substitution from sense codons to stop codons is not included in the ECM matrix, which makes the matrix block diagonal with a 61×61 matrix for coding codons and a 3×3 entries for substitutions between stop codons. Therefore, we will consider only substitutions between coding codons and regard the ECM matrix as 61×61. From the communication perspective, this mutation matrix describes channel transition probabilities **P**(*y*|*x*).

There is also another matrix in [4], which gives the actual count of substitutions observed. From this substitution count matrix **C**, we obtained the biological probability distribution of the codons as

(1) $$p_{x}=\frac{\underset{j}{\sum }C_{\text{ij}}}{\underset{i}{\sum }\underset{j}{\sum }C_{\text{ij}}}\;.$$

Thereafter, we combined the codons which encode for the same amino acid and computed the probability distribution of amino acids, denoted **p**_*a*_. Using this distribution, the to be preserved information content of the 64 codons representing the 20 amino acids can be computed as

(2) $$R_{20}=-\overset{20}{\underset{i=1}{\sum }}p_{a}(i)\underset{2}{\log}(p_{a}(i)).$$

According to Shannon’s channel coding theorem, a communication through a noisy channel of capacity *C* at an information rate of *R* is possible with an arbitrarily small probability of error, if *R*<*C*[10]. Hence, the channel capacity has to, at least, exceed the value of *R*_20_.

In communication systems, the channel capacity is determined by maximizing the mutual information between input (X) and output (Y) over the input probability distribution **p**_*x*_.

(3) $$C={\text{sup}}_{p_{x}}I(X;Y).$$

*I*(*X*;*Y*) is the mutual information which measures the mutual dependence between input and output distributions, and is defined as

(4) I(X;Y)=H(Y)-H(Y|X),

where *H*(*Y*) is the entropy of the codon distribution at the output of the ECM “channel”, and *H*(*Y*|*X*) is the conditional entropy, called prevarication or irrelevance.

However, in the system we are considering, the input distribution (i.e., probability distribution of codons) is not something to adjust. It is defined by nature. Therefore, we determine the channel capacity corresponding to the mutation “channel” matrix for a biological codon frequency obtained by Eq. (1). *H*(*Y*) is computed as

(5) $$H(Y)=-\overset{61}{\underset{i=1}{\sum }}p_{y_{i}}\underset{2}{\log}(p_{y_{i}}),$$

where $p_{y_{i}}$ is the output probability distribution of the *i*^*t* *h*^ codon. The conditional entropy *H*(*Y*|*X*) between input and output distribution of codons is computed as

(6) $$H(Y|X)=-\overset{61}{\underset{i=1}{\sum }}p(x_{i})\overset{61}{\underset{j=1}{\sum }}p(y_{j}|x_{i})\underset{2}{\log}p(y_{j}|x_{i}).$$

*p*(*y*_*j*_|*x*_*i*_) is the conditional probability between codons, which is given by the empirical codon mutation (ECM) matrix.

We now compute, what exponent of the ECM matrix would be needed to make the capacity just match the required rate obtained by Eq. (2). Hereto, we use the singular value decomposition (SVD) yielding

(7) $${\left[P(y|x)\right]}^{F}=U{\left(\Sigma \right)}^{F}V^{*},$$

where **U,V** are unitary matrices, **Σ** is a diagonal matrix with nonnegative real numbers in the diagonal, and *F* is an exponent to be fine-tuned. The value of the exponent is changed in steps from zero to one. A value of 1 means the original ECM matrix is used.

Moreover, we would like to find the optimum codon distribution by solving Eq. (3) and compare it with the biological distribution. For solving the optimization problem, the Arimoto-Blahut algorithm was employed [8,9]. The Arimoto-Blahut algorithm is an iterative numerical algorithm that monotonically converges to the capacity value. To compute the capacity, it is starting from any arbitrary input probability distribution **p**_*x*_ (usually uniform) and performs the following two steps until the algorithm converges. 

1. Compute a quantity related to the mutual information per input symbol

(8) $$c(x_{j}):=\exp\underset{k}{\sum }p(y_{k}|x_{j})\log\frac{p(y_{k}|x_{j})}{\sum_{j}p(x_{j})p(y_{k}|x_{j})}.$$

This results from a Lagrange multiplier step in [9].

2. Update the input probability distribution according to

(9) $$p(x_{j})=\frac{p(x_{j})c(x_{j})}{\sum_{j}p(x_{j})c(x_{j})}\;.$$

The termination criteria is based on the lower and upper bounds of the channel capacity,

(10) $$\log\left(\underset{j}{\sum }p(x_{j})c(x_{j})\right)\leq C\leq \log\left(\underset{x_{j}}{\max}c(x_{j})\right).$$

The iterations are terminated when the upper and lower bounds are equal up to a certain accuracy.

Once the optimized codon distribution is obtained using the Arimoto-Blahut algorithm, to note the similarity with the biological distribution, we applied the so called Kullback-Leibler divergence (*D*_*K* *L*_) [11]. *D*_*K* *L*_ is a quantitative measure of how similar a probability distribution *P* is to a model distribution *Q*, and is defined as

(11) $$D_{\text{KL}}(P||Q)=\underset{i}{\sum }P_{i}\underset{2}{\log}\frac{P_{i}}{Q_{i}}.$$

*D*_*K* *L*_ is non-negative and gives a zero result when the distributions are perfectly matched. Technically speaking, *D*_*K* *L*_ measures the average number of extra bits required (coding penalty) for using a code based on *Q* instead of *P*.

## Results and discussion

**Figure 1:**
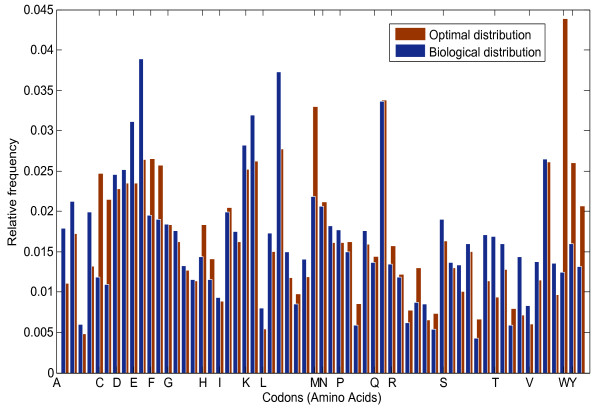
**Probability distribution of codons (Biological and Optimal).** The optimum codon distribution to maximize mutual information and the biological distribution of codons in the five vertebrates. Consecutive bins indicate that the codons belong to the same encoded amino acid (one letter symbol). The synonymous codons are arranged alphabetically.

The to be preserved information content of the amino acids, using the amino acid distribution and computed according to Eq. (2) is 4.1875 bit, which is less than the maximum value of log2(20)=4.3219 bit. Likewise, the required rate obtained by using the amino acid probability distribution provided by King & Jukes in [12], derived from 5492 residues of 53 vertebrate polypeptides is 4.2033 bit. Thus, it is reasonable to look for a capacity that is at least greater than 4.1875. Hence, using the biological codon distribution in the five vertebrates obtained by using Eq. (1), we stepwise reduced the exponent of the ECM matrix until it satisfies the rate requirement. Furthermore, we used the Arimoto-Blahut algorithm to find the optimal input probability distribution of the 61 codons to maximize the mutual information and compare it with the biological distribution of codons. The optimal capacity-achieving codon distribution and the observed biological codon distribution are both shown in Figure 1. The corresponding values are also tabulated in Table 1 and Table 2.

**Table 1:** Biological codon relative frequency

	**Codon**	**Frequency**	**Codon**	**Frequency**	**Codon**	**Frequency**	**Codon**	**Frequency**	
T	TTT	0.0191	TCT	0.0171	TAT	0.0132	TGT	0.0110	T
	TTC	0.0196	TCC	0.0160	TAC	0.0160	TGC	0.0119	C
	TTA	0.0085	TCA	0.0133	TAA	0.0003	TGA	0.0003	A
	TTG	0.0141	TCG	0.0043	TAG	0.0001	TGG	0.0125	G
C	CTT	0.0150	CCT	0.0176	CAT	0.0116	CGT	0.0054	T
	CTC	0.0173	CCC	0.0150	CAC	0.0144	CGC	0.0087	C
	CTA	0.0080	CCA	0.0178	CAA	0.0137	CGA	0.0062	A
	CTG	0.0373	CCG	0.0059	CAG	0.0337	CGG	0.0085	G
A	ATT	0.0175	ACT	0.0144	AAT	0.0182	AGT	0.0136	T
	ATC	0.0200	ACC	0.0160	AAC	0.0206	AGC	0.0191	C
	ATA	0.0094	ACA	0.0169	AAA	0.0282	AGA	0.0135	A
	ATG	0.0219	ACG	0.0059	AAG	0.0319	AGG	0.0118	G
G	GTT	0.0136	GCT	0.0200	GAT	0.0252	GGT	0.0115	T
	GTC	0.0138	GCC	0.0213	GAC	0.0246	GGC	0.0176	C
	GTA	0.0084	GCA	0.0179	GAA	0.0311	GGA	0.0184	A
	GTG	0.0265	GCG	0.0060	GAG	0.0389	GGG	0.0133	G
	T		C		A		G		

The codon relative frequency of the five vertebrates genomes (human, mouse, chicken, frog, and zebrafish) from the data presented by Schneider A., Cannarozzi G., and Gonnet G. [4].

**Table 2:** Calculated codon relative frequency

	**Codon**	**Frequency**	**Codon**	**Frequency**	**Codon**	**Frequency**	**Codon**	**Frequency**	
T	TTT	0.0257	TCT	0.0113	TAT	0.0207	TGT	0.0215	T
	TTC	0.0264	TCC	0.0150	TAC	0.0260	TGC	0.0247	C
	TTA	0.0097	TCA	0.0100	TAA	*	TGA	*	A
	TTG	0.0119	TCG	0.0066	TAG	*	TGG	0.0439	G
C	CTT	0.0118	CCT	0.0159	CAT	0.0141	CGT	0.0073	T
	CTC	0.0150	CCC	0.0162	CAC	0.0183	CGC	0.0129	C
	CTA	0.0054	CCA	0.0161	CAA	0.0144	CGA	0.0077	A
	CTG	0.0277	CCG	0.0085	CAG	0.0337	CGG	0.0065	G
A	ATT	0.0162	ACT	0.0071	AAT	0.0160	AGT	0.0130	T
	ATC	0.0205	ACC	0.0128	AAC	0.0212	AGC	0.0163	C
	ATA	0.0088	ACA	0.0093	AAA	0.0251	AGA	0.0157	A
	ATG	0.0330	ACG	0.0079	AAG	0.0261	AGG	0.0122	G
G	GTT	0.0096	GCT	0.0132	GAT	0.0234	GGT	0.0114	T
	GTC	0.0114	GCC	0.0172	GAC	0.0228	GGC	0.0162	C
	GTA	0.0060	GCA	0.0110	GAA	0.0235	GGA	0.0183	A
	GTG	0.0260	GCG	0.0048	GAG	0.0263	GGG	0.0126	G
	T		C		A		G		

The codon relative frequency that maximizes the mutual information between input and output and yielding a capacity close to what is required for preserving the information content of amino acids. An exponential factor of 0.26 is applied to the ECM matrix.

**Figure 2:**
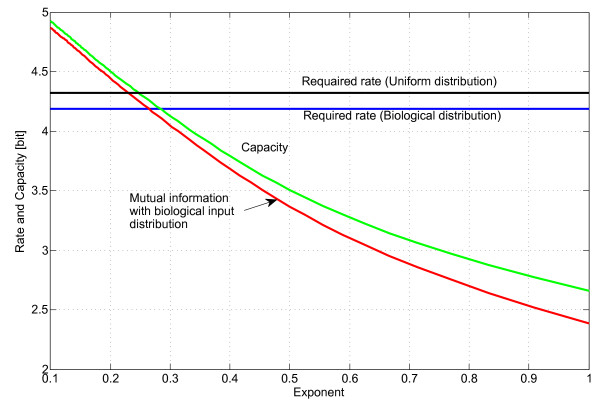
**Capacity as a function of an exponential factor.** The required rate for error-free transmission and the achievable capacity are plotted as a function of the exponent of the ECM matrix.

The capacity obtained by optimizing the codon distribution, the mutual information based on the observed biological codon distribution, and the required rate are shown together in Figure 2. When the exponent of the ECM matrix is reduced, the output codon distribution changes and the prevarication *H*(*Y*|*X*) will be smaller. As a result, the capacity increases. The maximal exponent which satisfies the rate requirement of 4.1854 bit for an error-free “transmission” using the biological codon frequency is found to be ≈ 0.26. At the same exponent, the optimized “channel” capacity is 4.2586 bit. It can also be seen that the capacity curve is very close to the one found by using the biological codon distribution. This indicates that the biological probability distribution is almost optimally “chosen” to achieving the capacity of the “channel”.

It is not surprising that the exponent is not one, since the matrix was obtained comparing five different vertebrate DNAs, the times corresponding to time spans between 40 M – 350 M years. However, the exponent is not extremely small, which indicates that the matrix is at least roughly in agreement with information-theoretic calculations. One may also see this as an argument to recompute the matrix using the obtained coefficient.

To see how well the biological and the optimized codon distributions agree, we applied the Kull-back–Leibler divergence (*D*_*K* *L*_) and obtained a value of 0.0926 bit, which is not a very small difference (comparable with the *D*_*K* *L*_ of two Gaussians of equal mean and a variance differing by a factor of two) but still, similarities are obvious. Both of the probability distributions satisfy the rate requirement of 4.1875 bit. In addition, the distribution among synonymous codons is very similar. To mention one example, codons encoding Alanine (A) in decreasing order of abundance, is GCC, GCT, GCA, and GCG, for both the biological and the capacity-achieving distributions.

## Conclusion

From the so-called empirical codon substitution matrix (ECM), a mutation probability matrix, we derived the capacity when regarding the matrix as a communication channel. We found that an exponent of 0.26 would lead to a capacity of 4.1875 bit that is at least required to preserve the genetic information represented by the 20 amino acids encoded by 64 codons. Additionally, for the desired channel capacity, we have presented the optimal codon distribution found by searching the distribution that maximizes the mutual information starting from a random initialization. A comparison of the biological distribution with the optimized codon distribution shows that the two distributions are not too similar. However, the biological distribution is not too far from the capacity-achieving distribution in terms of “channel” capacity, which indicates that the biological distribution is well “chosen”. Additionally, the optimal codon distribution has preserved the relative abundance of synonymous codons. We concluded that the ECM as a channel is not too far from what would be expected following information theoretic arguments although it was derived from 5 different species.

## Competing interests

We have no competing interests.

## Authors’ contributions

AM: Original computations and first version of the paper for first review. DN: Correcting computations and major updates of the paper according to the wishes of the reviewers. WH: Supervisor, original idea, checking results, and proof-reading. All authors read and approved the final manuscript.
